# Clomiphene citrate ameliorated lead acetate-induced reproductive toxicity in male Wistar rats

**DOI:** 10.5935/1518-0557.20190038

**Published:** 2019

**Authors:** Oyeyemi A. Wahab, Anyanwu C. Princely, Akinola A. Oluwadamilare, Daramola O. Oore-oluwapo, Alli O. Blessing, Ehiaghe F. Alfred

**Affiliations:** 1 Department of Physiology, Igbinedion University, Okada, Edo State, Nigeria; 2 Department of Physiology, University of Medical Sciences, Ondo-City, Ondo State, Nigeria; 3 Department of Haematology, Igbinedion University, Okada, Edo State, Nigeria

**Keywords:** 17β-hydroxysteroid dehydrogenase, clomiphene citrate, gonadotropins, lead acetate, sperm count

## Abstract

**Objective:**

The current study investigated the effects of clomiphene citrate on the hypothalamic-pituitary-testicular axis, steroidogenesis, sperm parameters, and testicular antioxidant enzyme activity of male Wistar rats submitted to lead acetate (Pb)-induced reproductive toxicity.

**Methods:**

Twenty adult male Wistar rats were divided into four groups of equal size as follows: Control; Clomid (0.35 mg/kg); Pb (10 mg/kg); and Clomid + Pb. Serum levels of follicle stimulating hormone (FSH), luteinizing hormone (LH), testosterone, testicular 17-β hydroxysteroid dehydrogenase (17-β HSD) activity, androgen receptors, catalase activity, superoxide dismutase (SOD), malondialdehyde (MDA), sperm motility, viability, counts and morphology were estimated after oral administration of Clomid and/or lead acetate for 35 consecutive days. Data were analyzed using ANOVA at *p*<0.05.

**Results:**

Lead acetate significantly decreased (*p*<0.05) serum LH and testosterone levels, testicular 17β-HSD activity, androgen receptor expression, sperm motility, viability, counts, catalase activity, and SOD when compared with controls. Abnormal sperm morphology and MDA were significantly increased (*p*<0.05) in the Pb group compared with controls. Clomid co-administrated with lead acetate significantly increased (*p*<0.05) serum LH, testosterone levels, testicular 17β-HSD, androgen receptor expression, sperm motility and viability when compared with the group given lead acetate.

**Conclusions:**

The present study suggests that clomiphene citrate may stimulate testicular testosterone synthesis, sperm motility and viability via luteinizing hormone in a context of lead acetate-induced reproductive toxicity.

## INTRODUCTION

Many environmental and occupational chemicals are harmful to reproductive function and fertility (Cherry *et al.,* 2008; Taskinen *et al.,* 2011). Lead stands out as an environmental and occupational chemical agent. This heavy metal occurs naturally in the environment and from activities such as burning fossil fuels, mining, and manufacturing (Gabby, 2006). It is also present in various domestic and industrial applications such as the production of ammunition, cosmetics, glass pigments, lead-acid batteries, metal products (solder and pipes), oxides for paints, and devices to shield against X-rays (Gabby, 2006). It may be present as a pollutant in different sources such as contaminated food, lead water pipes, unsanitary food preservation, industrial pollution, road traffic, paint, cosmetics, and drinking water (López-Carrillo *et al.,* 1996). It has been well documented that lead impairs the reproductive function of experimental animals and humans through endocrine disruption (Biswas & Ghosh, 2004; Hernández-Ochoa *et al.,* 2005; Jackie *et al.,* 2011) and depletion of antioxidant reserves (Gorbel *et al.,* 2002; Elgawish & Abdelrazek, 2014).

Clomiphene citrate (Clomid) is an orally active non-steroidal fertility drug (Patankar *et al*., 2007). It is a selective estrogen receptor inhibitor in the hypothalamus. It acts by inhibiting negative feedback of estrogen on gonadotropin release, leading to the up-regulation of the hypothalamic-pituitary-gonadal axis (Tenover & Bremner, 1991; Guay *et al.,* 1995). It ultimately stimulates testosterone and sperm production. Patankar *et al*. (2007) and Katz *et al.* (2012) reported increased levels of serum follicle stimulating hormone (FSH), luteinizing hormone (LH), testosterone, and spermatogenesis in infertile and hypogonadal men treated with Clomiphene citrate.

This study was designed to investigate the effects of clomiphene citrate on lead acetate-induced reproductive toxicity in male Wistar rats.

## MATERIALS AND METHODS

### Drug and Chemicals

The clomiphene citrate (Clomid) used in this study was a product of Doppel farmaceutici S.r.l., Via Martiri delle Foibe, Italy. Only analytical grade chemicals were used in this study.

### Animals

Fourteen-week-old male Wistar rats with a mean weight of 190±5.8g were used in this study. The rats were procured and kept in the animal house at Igbinedion University, Okada, Edo State, Nigeria. The animals were fed with commercially prepared pelletized mash, and had free access to water. They were housed under standard laboratory conditions and handled in accordance with the guidelines set out by the National Institutes of Health (NIH, 1988) and the National Research Council (NRC, 1996).

### Experimental Design

Twenty male Wistar rats were randomly divided into four groups of equal size as follows: Control; Clomid (0.35 mg/kg Clomid); Pb (10 mg/kg lead acetate); and Clomid + Pb (0.35mg/kg Clomid plus 10 mg/kg lead acetate). Clomid and lead acetate dosages were based on the reports by Patankar *et al*. (2007) and Hsu *et al.* (1997), respectively.

The rats in the treatment groups were given oral Clomid and/or lead acetate daily for 35 days. Each was anesthetized with an intraperitoneal injection of sodium thiopental (50 mg/kg) on day 36. Serum follicle stimulating hormone, luteinizing hormone, and testosterone levels were measured. Their testes and epididymes were harvested. The right testis was homogenized in phosphate buffer saline (PBS), and the supernatant was used for the estimation of testicular 17β-hydroxysteroid dehydrogenase (17β-HSD), malondialdehyde, catalase, and superoxide dismutase activity. The left testis was fixed in Bouins' solution for testicular histology and immunohistochemistry staining of androgen receptors. Sperm in the cauda epididymis was analysed.

### Hormone Analysis

Serum follicle stimulating hormone (FSH), luteinizing hormone (LH), and testosterone levels were measured using the ELISA method. The assay was carried out according to the instructions in the Calbiotech ELISA kit manual (Chen *et al.,* 1991; Heinonen, 1991; Qiu *et al.,* 1998; Rose, 1998; Ulloa-Aguirre & Timossi, 1998).

### Testicular 17β-hydroxysteroid Dehydrogenase Activity Assay

Testicular 17β-hydroxysteroid dehydrogenase (HSD) activity was measured according to the method described by Talalay (1962). The homogenised testes supernatant (1 mL) was mixed with an equal volume of 440 µmol sodium pyrophosphate buffer (pH 10.2), 40 µL of 0.3 µmol testosterone, and 960 µL of 2.5 % bovine serum albumin, bringing the incubation mixture to a total volume of 3 mL. Enzyme activity was measured after the addition of 1.1 µmol nicotinamide adenine dinucleotide (NAD) to the incubated mixture in a spectrophotometer cuvette at 340 nm against a blank (without NAD). One unit of enzyme activity is equivalent to a change in absorbance of 0.001/min at 340nm.

### Testicular Histology and Immunohistochemistry Staining for Androgen Receptors

Five-micrometre sections of the testes were mounted on slides to stain androgen receptors by immunohistochemistry. The sections were dewaxed, rehydrated, and autoclaved at 120 ºC for 10 minutes in 10 mM citrate buffer (pH 6.0). After washing with phosphate buffer saline (PBS), endogenous peroxidase was blocked using 0.3% hydrogen peroxide in methanol for 15 minutes. The slides were rewashed in PBS and blocking was performed by adding blocking buffer. Then they were incubated for 30 minutes at room temperature. Primary monoclonal and polyclonal antibodies for androgen receptors were added after dilution by PBS (2 µg/mL) and incubated for 30 minutes. The slides were washed three times for 3 minutes each with PBS. Biotinylated polyvalent secondary antibody was applied to tissue sections and incubated for 30 minutes. The slides were washed three times for 3 minutes each with wash buffer. Metal-Enhanced 3,3'-diaminobenzidine (DAB) substrate working solution was added to the tissue and incubated 10 minutes for visibility of the reaction. The slides were washed two times for 3 minutes each with wash buffer and counterstained with hematoxylin stain (Ramos-Vara, 2011). Photomicrographs of the slides were taken under a light microscope at 200x magnification with an Omax 10.0MP digital camera.

### Sperm Analysis

### Sperm Motility

Sperm motility and viability assessment were done immediately after the rats were anesthetized. The right epididymis was immediately excised with care to minimize blood adulteration and placed into a pre-warmed (37 ºC) Petri dish containing two mL of phosphate buffer saline solution (pH 7.4). The caudal portion was punctured twice with the tip of a scalpel blade to release sperm, commencing a 3-minute "swim-out" period. After the swim-out, the dish was gently swirled, and a drop of sperm suspension was put on a warmed microscope slide and covered with a coverslip. It was then observed at 400x magnification on an optical microscope. Sperm motility was assessed based on the guidelines set out by the WHO (Tannenbaum *et al*., 2003; WHO, 2010).

### Sperm Viability

An equal volume (10 µ) of sperm suspension and eosin-nigrosin staining solution were placed on a microscope slide, which was then covered with a coverslip. The slides with sperm samples were air-dried and observed on a light microscope at a magnification of 1,000x. Dead (stained) and living sperm (not stained) were counted and expressed as percentages (Björndahl *et al*., 2003). 

### Sperm Count

The left caudal portion of the epididymis was minced in a petri dish containing 2 mL of deionized water to form a sperm suspension. The sperm suspension was diluted in sodium bicarbonate-formalin solution at a ratio of 1:20. A drop of diluted sperm mixture was transferred to an improved Neubauer hemocytometer chamber and the sperm cells were counted in 2 square mm under the microscope. Spermatozoa counts were calculated and expressed in million/mL (Tannenbaum *et al*., 2003; WHO, 2010).

### Sperm Morphology

Two drops of sperm suspension were placed on a microscope slide and a thin sperm smear was then made. The slide was then air-dried. The sperm smears were stained with eosin-nigrosin and examined for abnormal sperm morphology on a microscope at a magnification of 1,000x. A total of 100 sperms per sample were evaluated and abnormal sperm morphology was expressed as a percentage (Tannenbaum *et al*., 2003).

### Testicular Oxidant and Antioxidant Enzyme Assay

### Malondialdehyde

Malondialdehyde was used to assess the level of lipid peroxidation according to the method described by Buege & Aust (1978). The reaction mixture was made up of 1.0 mL homogenised testicular supernatant with 2.0 mL of trichloroacetic acid-thiobarbituric acid-hydrochloric acid reagent (TCA-TBA-HCl). The mixture was shaken and heated in a boiling water bath for 20 minutes, then cooled, centrifuged, and the color developed in the supernatant was measured at 535 nm. The concentration of MDA was calculated using the extinction coefficient of MDA-TBA complex, which is 1.56×10^5^ M^-1^ cm^-1^, and the results were expressed as nM/mg tissue.

### Catalase Activity

Catalase activity was assayed according to the method described by Cohen *et al*. (1970). Homogenized testes (0.5 mL) was mixed with an equal volume of 30M hydrogen peroxide, 1ml of 6M H_2_SO_4_, and 7 ml of 0.01M of potassium permanganate. Absorbance was read at 480nm within 30 to 60 seconds against distilled water. The result was expressed in µM/mg protein.

### Superoxide Dismutase (SOD) Activity

Testicular SOD activity was determined based on the principle of inhibition of epinephrine autoxidation in an alkaline medium at 480 nm in a UV spectrophotometer (Misra & Fridovich, 1972). SOD activity was expressed in arbitrary units considering the inhibition of autoxidation, as 1 unit of SOD activity (mU/mg tissue).

### Digital Image Analysis/quantification of Androgen Receptors using ImageJ

The photomicrographs were trained by selecting a Region of Interest (ROI) through a rectangular tool from the immunohistochemistry toolbox of software package ImageJ (version 1.49, National Institutes of Health, Bethesda, MD, USA, http://rsb.info.nih.gov/ij/). The read mode button of the toolbox was set to read the default model H-DAB.txt for brown color detection. Once the color detection model (for example, the H-DAB. txt) was read, the segmentation and quantification of androgen receptor nuclei were estimated. Quantification consists of counting the positive nuclei and oval fitted nuclei segmentation (Shu *et al.*, 2014). 

### Statistical Analysis

Data were analysed statistically using analysis of variance (ANOVA), followed by least significant difference (Post Hoc test) and results were expressed as mean ± Standard Error of Mean (mean ± SEM). Differences between mean values were considered significant at *p*<0.05.

## RESULTS

### Serum Gonadotropin and Testosterone Levels

Table 1 shows the results for serum testosterone and gonadotropin levels in the male Wistar rats co-treated with Clomid and lead acetate. Serum testosterone levels were significantly reduced (*p*<0.05) in the Pb and Clomid + Pb groups when compared with controls, while significant increases (*p*<0.05) were observed in the group treated with Clomid plus Pb when compared with the Pb group. Serum LH levels were significantly increased *p*(<0.05) in the Clomid group and a significant decrease (*p*<0.05) was noticed in the Pb group when compared with controls, while significant increases (*p*<0.05) were observed in the Clomid + Pb group relative to the Pb group.

**Table 1:** Effects of clomid on testosterone, follicle stimulating hormone, and luteinizing hormone in male Wistar rats exposed to lead acetate

Group (n=5)	Follicle stimulating hormone (mIU/mL)	Luteinizing hormone (mIU/mL)	Testosterone (ng/mL)
Control	2.72±0.04	3.54±0.07	2.90±0.26
Clomid	2.83±0.05	4.22±0.21*	3.54±0.35
Pb	2.64±0.22	3.08±0.15*	0.40±0.04*
Clomid + Pb	3.07±0.25	3.58±0.10^+^	1.57±0.22*^,^+

Values expressed as mean ± SEM

*,^+^ *p*<0.05 shows a significant difference when compared with controls and Pb, respectively.

### Testicular 17β-hydroxysteroid Dehydrogenase Activity

**Figure 1:**
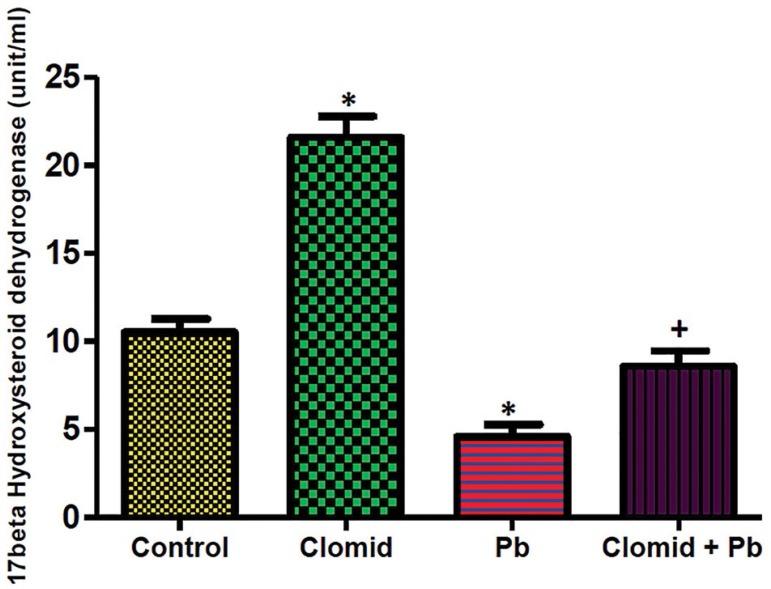
Effect of clomid on 17βhydroxysteroid dehydrogenase activity in male Wistar rats exposed to lead acetate. Bars show mean ±SEM *,+ *p*<0.05 was considered significant relative to control and Pb groups, respectively.

Figure 1 shows that 17β-hydroxysteroid dehydrogenase activity was significantly increased (*p*<0.05) in the Clomid group when compared with controls. The activity of this enzyme was significantly reduced (*p*<0.05) in the Pb and Clomid + Pb groups when compared with controls, while there was a significant increase (*p*<0.05) in the Clomid + Pb group relative to the Pb group.

### Testicular Androgen Receptor Expression

**Figure 2:**
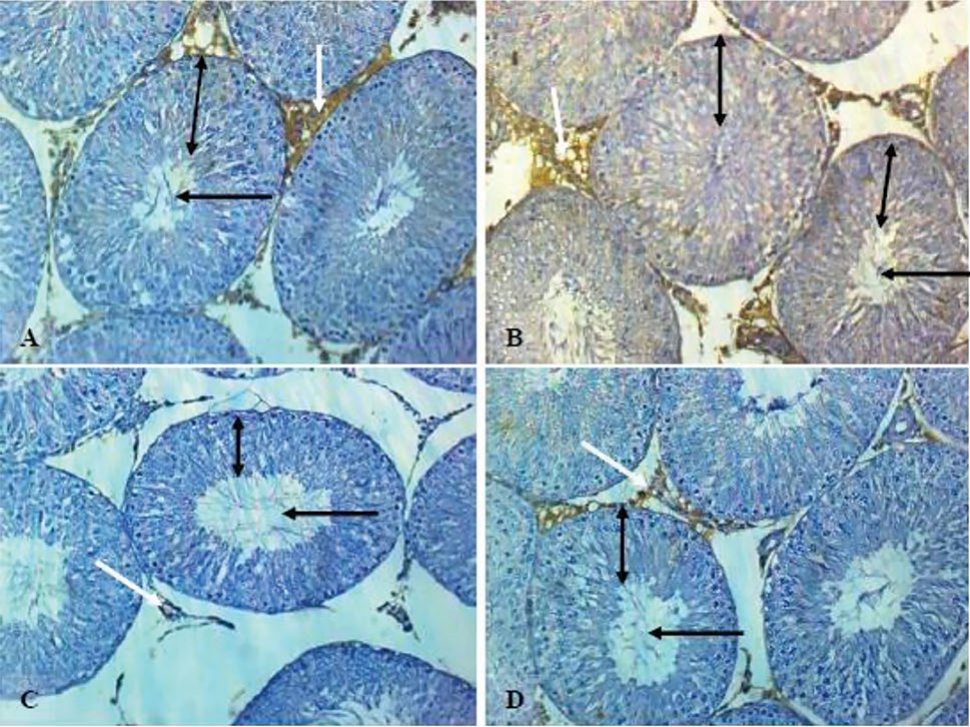
Effect of clomid on testicular histology and androgen receptor (AR) expression in male Wistar rats exposed to lead acetate (A): Control group showed moderate AR expression (white arrow), normal seminiferous tubules with the presence of spermatozoa strand in the lumen (black arrow) and normal stratification of germ cell layer (spanned). (B): Clomid group showed strong AR expression (white arrow), normal seminiferous tubules and stratification of spermatogenic cells (spanned) with the presence of whorl spermatozoa (black arrow). (C): Pb group showed mild AR expression (white arrow), widened interstitial space and lumen (black arrow) were observed with sloughing and arrest of germ cell layer stratification (spanned). (D): Clomid + Pb group showed moderate AR expression (white arrow), some normal germ cell layers (spanned) with a moderate lumen (black arrow) and normal seminiferous tubules.

**Figure 3:**
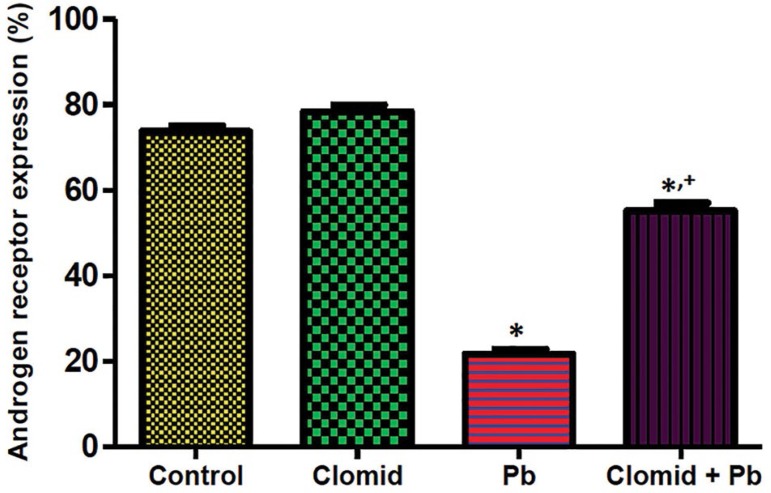
Quantification of testicular androgen receptor expression in male Wistar rats co-treated with clomid and lead acetate. Bars show mean±SEM *,+ *p*<0.05 was considered significant relative to control and Pb groups, respectively.

Figures 2 and 3 show the expression of testicular androgen receptors in male Wistar rats co-treated with Clomid and lead acetate. Androgen receptor expression was significantly reduced (*p*<0.05) in the Pb group when compared with controls, while significant increases (*p*<0.05) were observed in male Wistar rats in the Clomid + Pb group when compared with the Pb group.

### Sperm Motility, Viability, Counts, and Abnormal Morphology

**Figure 4:**
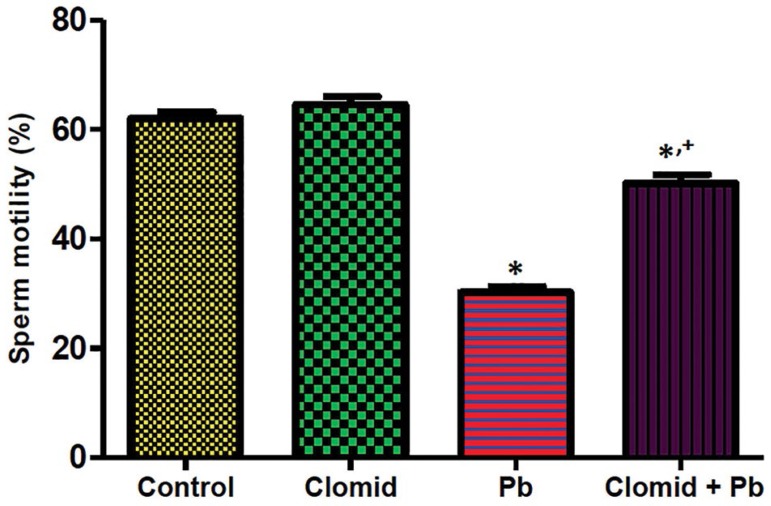
Effect of clomid on sperm motility in male Wistar rats exposed to lead acetate. Bars show mean±SEM *,+*p*<0.05 was considered significant relative to control and Pb groups, respectively.

**Figure 5:**
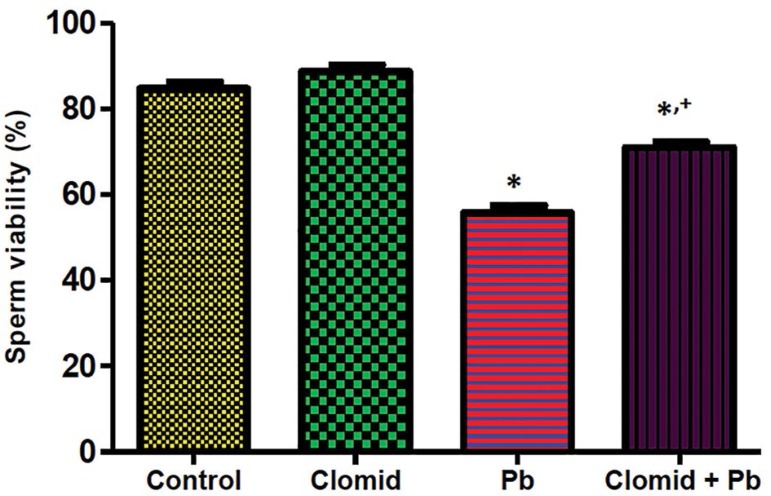
Effect of clomid on sperm viability in male Wistar rats exposed to lead acetate. Bars show mean±SEM *,+ *p*<0.05 was considered significant relative to control and Pb groups, respectively.

Figures 4 and 5 show that sperm motility and viability were significantly decreased (*p*<0.05) in the male Wistar rats treated with lead acetate when compared with controls, while a significant increase (*p*<0.05) was observed in the rats co-treated with Clomid and lead acetate when compared with the Pb group.

**Figure 6:**
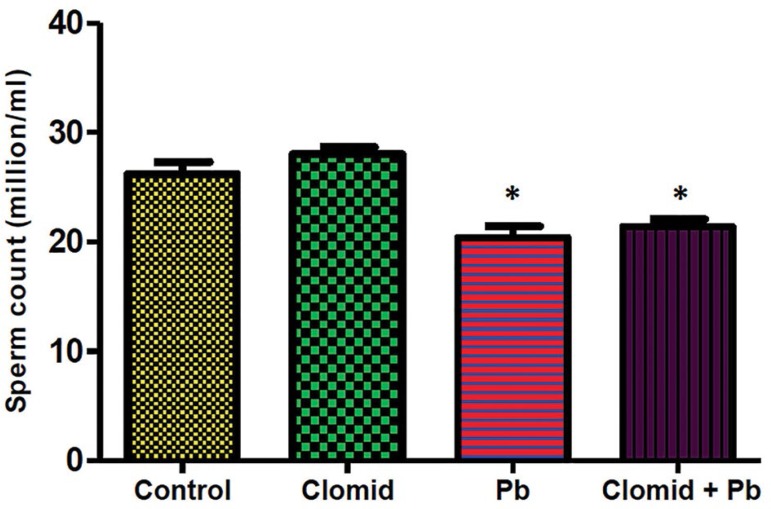
Effect of clomid on sperm counts of male Wistar rats exposed to lead acetate. Bars show mean±SEM *p<0.05 was considered significant relative to the control group.

**Figure 7:**
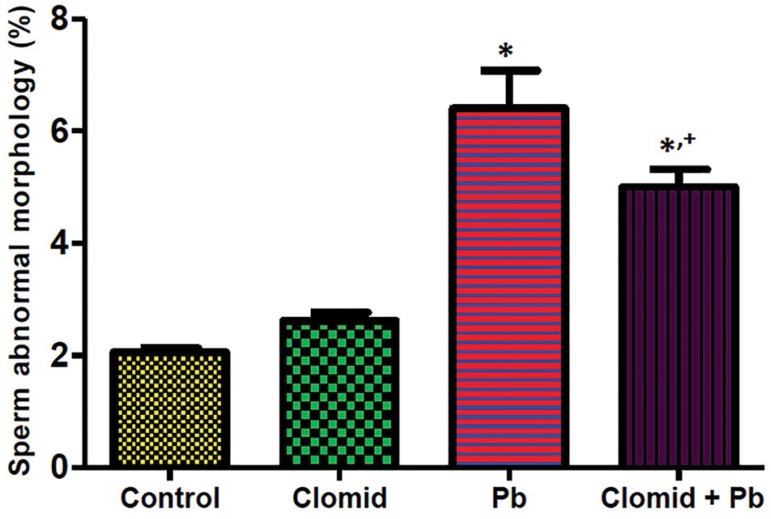
Effect of clomid on sperm abnormal morphology in male Wistar rats exposed to lead acetate. Bars show mean±SEM *,+ *p*<0.05 was considered significant relative to control and Pb groups, respectively.

Figure 6 shows that sperm counts were significantly decreased (*p*<0.05) in the Pb and Clomid + Pb groups when compared with controls. Figure 7 shows that abnormal sperm morphology was significantly increased (*p*<0.05) in the male Wistar rats in the Pb and Clomid + Pb groups when compared with controls, while a significant decrease (*p*<0.05) was observed in the Clomid + Pb group when compared with the Pb group.

### Oxidant and Antioxidant Enzyme Activities

Table 2 shows the results of testicular malondialdehyde, catalase, and superoxide dismutase activities. Testicular malondialdehyde levels were significantly increased (*p*<0.05) in the Pb and Clomid + Pb groups when compared with controls. Testicular catalase and superoxide dismutase activities were significantly decreased (*p*<0.05) in the Pb and Clomid + Pb groups when compared with controls. 

**Table 2:** Effects of clomid on testicular oxidant and antioxidant enzymes activity in male Wistar rats exposed to lead acetate

Group (n=5)	Malondialdehyde (nM/mg tissue)	Catalase (µM/mg tissue)	Superoxide dismutase (mU/mg tissue)
Control	0.50±0.02	48.40±2.31	76.10±2.45
Clomid	0.42±0.09	47.62±3.25	74.02±4.30
Pb	0.81±0.22*	26.34±2.60*	41.00±3.42*
Clomid + Pb	0.66±0.04*	30.20±1.58*	45.57±2.22*

Values expressed as mean±SEM,

* *p*<0.05 shows a significant difference when compared with controls and Pb, respectively.

## DISCUSSION

Lead acetate is an established male reproductive toxicant. It usually disrupts the hypothalamic-pituitary-testicular axis, steroidogenesis, and spermatogenesis, in addition to increasing the generation of reactive oxygen species (Ait-Hamadouche *et al.,* 2013a; Elgawish & Abdelrazek, 2014; Al-Masri, 2015). This study examined the possible effects of clomiphene citrate on the hypothalamic-pituitary-testicular axis, steroidogenesis, sperm parameters, and testicular antioxidant enzymes activity of male Wistar rats exposed to lead acetate.

Follicle stimulating hormone normally acts on Sertoli cells to trigger the formation of spermatogonial cells, testosterone-binding protein, and spermatogenesis in the testes (Wilhelm *et al*., 2007; Garu *et al*., 2011). There are contradicting reports on the effects of lead on serum FSH levels. Some studies reported decreased serum FSH levels in Wistar rats (Biswas & Ghosh, 2004; Al-Masri, 2015), while Petrusz *et al.* (1979) and Ng *et al.* (1991) reported increases in serum FSH levels. Our study showed no alteration in serum FSH levels after oral administration of lead acetate for thirty-five days in male Wistar rats. This observation is in agreement with Pinon-Lataillade *et al*. (1995). Clomid increased FSH, but not significantly in the rats given lead acetate.

Luteinizing hormone is a prominent gonadotropin that stimulates and regulates testosterone synthesis in Leydig cells. Lead has been reported to reduce serum LH levels in male Wistar rats (Biswas & Ghosh, 2004; Ait-Hamadouche *et al*., 2013b; Al-Masri, 2015). The exposure of male Wistar rats to lead acetate in the present study reduced serum LH levels. This may have occurred through the disruption of the hypothalamic-pituitary axis (Hernández-Ochoa *et al.,* 2005; Elgawish & Abdelrazek, 2014).

Alterations in luteinizing hormone-releasing hormone (LHRH) gene expression (Gore, 2001) by an endocrine disruptor such as lead may be another possible mechanism responsible for the observed reduction in serum LH of lead acetate-exposed rats. Luteinizing hormone-releasing hormone secretion by the hypothalamus is responsible for regulating LH formation and secretion by the anterior pituitary through a negative feedback mechanism. In our study, LH decreases in lead acetate-exposed rats was improved with the administration of Clomid. Although luteinizing hormone-releasing hormone was not estimated in this study, Clomid is known for stimulating the release of this hormone (Rönnberg *et al*., 1985; Ribeiro & Abucham, 2009), which in turn may stimulate gonadotropes in the anterior pituitary gland to synthesise and release LH into the bloodstream.

Furthermore, male Wistar rats exposed orally to lead acetate had decreased serum testosterone levels in the current study. Lead is known to suppress steroidogenesis and decrease serum testosterone levels (Hernández-Ochoa *et al.,* 2005; Elgawish & Abdelrazek, 2014). Luteinizing hormone is the primary signal for initiating/stimulating steroidogenesis in Leydig cells. In our study, the observed reduction in serum testosterone levels of lead acetate-exposed rats was accompanied by decreases in LH. Thus, it can be inferred that the decreases in serum LH levels in lead acetate-exposed rats may hinder steroidogenesis in Leydig cells and effect the observed reduction in serum testosterone levels. Clomid administration increased the serum testosterone levels that were reduced in lead acetate-exposed rats. This observation may be linked to the stimulatory effect of Clomid on the hypothalamus to release luteinizing hormone-releasing hormone, thus stimulating the secretion of gonadotropins in the anterior pituitary gland and the production of LH, which usually initiates steroidogenesis in Leydig cells. The anti-estrogenic and aromatase inhibition properties of Clomid (Rönnberg *et al*., 1985; Ribeiro & Abucham, 2009) may also explain the increased serum testosterone levels observed.

17β-hydroxysteroid dehydrogenase is an essential enzyme in the steroidogenesis pathway that is accountable for catalysing the conversion of androstenedione to testosterone in Leydig cells. The present study showed that lead acetate reduced testicular 17β-hydroxysteroid dehydrogenase activity, an effect that most likely contributed and affected the observed reduction in serum testosterone levels. This observation is consistent with previous reports, in which lead directly inhibited steroidogenesis by reducing 17β-hydroxysteroid dehydrogenase activity (Biswas & Ghosh, 2004). The possible mechanism of lead in reducing 17β-hydroxysteroid dehydrogenase activity in the current study may be a result of the parallel reduction in LH, since LH signals the initiation of steroidogenesis. Hence, the decline in testicular 17β-hydroxysteroid dehydrogenase activity in the lead acetate-exposed rats may explain the observed decrease in serum testosterone levels in the rats treated with lead acetate in this study. The recorded decrease in testicular 17β-hydroxysteroid dehydrogenase activity in lead acetate-exposed rats was ameliorated with the administration of Clomid. Clomid usually stimulates LH secretion and release. The effects of luteinizing hormone in the initiation of steroidogenesis may also promote the activity of 17β-hydroxysteroid dehydrogenase (Rönnberg *et al*., 1985; Ribeiro & Abucham, 2009).

The present study also observed that lead acetate caused a marked reduction in testicular androgen receptor expression. Lead is a known endocrine disruptor with estrogenic properties that may be responsible for antagonising androgen receptor expression in male Wistar rats (Mattison, 1983; Naz, 1999; Elgawish & Abdelrazek, 2014). The reduction in testicular androgen receptor expression seen in lead acetate-exposed rats may also be accounted for by a parallel decline in serum testosterone levels. Testosterone is known to regulate androgen receptor expression through 5α-reductase. The co-administration of Clomid with lead up-regulated testicular androgen receptor expression, a finding possibly related to the aromatase-inhibiting property of Clomid that may prevent the conversion of androgen into estrogen (Rönnberg *et al*., 1985; Ribeiro & Abucham, 2009).

The results of the current study showed a reduction in sperm motility, viability, and counts, while abnormal sperm morphology such as swelled head, coiled tail, and detached head observed after oral administration of lead acetate. This observation was in agreement with previous reports (Hsu *et al.,* 1997; Naha *et al*., 2005; Hernández-Ochoa *et al*., 2005). The observed impacts in these sperm parameters may be linked to the direct toxic effects of lead acetate on sperm cells, increased generation of reactive oxygen species (Kasperczyk *et al*., 2004), and disruption of the hypothalamic-pituitary-gonadal axis. Reactive oxygen species are known to inhibit the production of sulfhydryl antioxidants, inhibit enzyme reactions, damage nuclei, and initiate lipid peroxidation in cell membranes required to give the plasma membrane the fluidity essential for sperm motility (Sanocka & Kurpisz, 2004). The observed reduction in serum luteinizing hormone and testosterone levels of rats given lead acetate in this study may also be implicated in the reduction of sperm motility, viability, and counts.

Clomid enhances LH and FSH secretion from the anterior pituitary (Chehab *et al*., 2015; Ring *et al.,* 2016). Increasing LH had been reported to facilitate both testosterone production and spermatogenesis (Chehab *et al*., 2015; Ring *et al.,* 2016). The stimulatory effect of Clomid on serum LH and testosterone levels in lead acetate-exposed rats in the current study did not improve sperm counts or sperm morphology. This may have occurred due to the generation of reactive oxygen species and the direct toxic effects of lead acetate on primordial germ cells, spermatocytes, and spermatozoa (Elgawish & Abdelrazek, 2014). Testicular histology in the current study may also be used to support the observed reduction in sperm counts of rats co-treated with Clomid and lead acetate with slight sloughing, arrest of germ cell layer stratification, and lumen widening. Nevertheless, sperm motility and viability in lead acetate-exposed rats improved when Clomid was administered.

Catalase and superoxide dismutase are endogenous antioxidant enzymes that scavenge free radicals. They act synergistically to remove superoxide anions generated by NADPH-oxidase in the cells and play an essential role in decreasing oxidative stress and membrane lipid peroxidation. Higher polyunsaturated fatty acid levels in the testes may subject it to oxidative stress and damage (Acharya *et al*., 2006). Results of the current study showed a reduction in the testicular catalase and superoxide dismutase activity of lead acetate-exposed rats. The results of the present study also showed an increase in malondialdehyde concentration in the testes of rats treated with lead acetate. The observed increased in malondialdehyde concentrations in the testes of rats treated with lead acetate indicates increased lipid peroxidation, since malondialdehyde results from the breakdown of polyunsaturated fatty acids and is considered a biomarker of lipid peroxidation. Hence, the observed reduction in catalase and superoxide dismutase activity together with the increase in malondialdehyde concentration in lead acetate-exposed rats serve as indication of the generation of free radicals and oxidative stress (Kasperczyk *et al*., 2004). This result supports previous reports in which lead acetate exposure induced oxidative stress due to the generation of free radicals and reactive oxygen species (Gorbel *et al.,* 2002; Kasperczyk *et al*., 2004; Elgawish & Abdelrazek, 2014).

Clomiphene citrate administration in lead acetate-exposed rats did not affect catalase and superoxide dismutase activity or malondialdehyde concentrations. This observation suggests that clomiphene citrate may not prevent lipid peroxidation, free radical generation, or oxidative stress induced by lead acetate.

This study suggests that clomiphene citrate may stimulate the testicular synthesis of testosterone, sperm motility and viability via luteinizing hormone in rats with lead acetate-induced reproductive toxicity. Decreases in sperm counts and endogenous antioxidants and increased lipid peroxidation pointed out that maximum safety precautions must be in place to prevent exposure to lead acetate. The observed stimulatory action of clomiphene citrate in this study may be temporary, since lead acetate disrupts the endocrine system through the generation of reactive oxygen species and the depletion of endogenous antioxidants. Hence, antioxidant supplementation combined with clomiphene citrate may be necessary to treat human infertility caused by exposure to heavy metals.

## References

[r1] Acharya UR, Mishra M, Tripathy RR, Mishra I (2006). Testicular dysfunction and antioxidative defense system of Swiss
mice after chromic acid exposure. Reprod Toxicol.

[r2] Ait-Hamadouche N, Nesrine S, Abdelkeder A (2013). Lead toxicity and hypothalamic-pituitary-testicular
axis. Not Sci Biol.

[r3] Ait-Hamadouche N, Sadi N, Kharoubi O, Slimani M, Aoues A (2013). The protective effect of vitamin E against genotoxicity of lead
acetate intraperitoneal administration in male rat. Not Sci Biol.

[r4] Al-Masri SA (2015). Effect of pumpkin oil and vitamin E on lead-induced testicular
toxicity in male rats. J Anim Plant Sci.

[r5] Biswas NM, Ghosh P (2004). Effect of lead on male gonadal activity in albino
rats. Kathmandu Univ Med J (KUMJ).

[r6] Björndahl L, Söderlund I, Kvist U (2003). Evaluation of the one-step eosin-nigrosin staining technique for
human sperm vitality assessment. Hum Reprod.

[r7] Buege JA, Aust SD (1978). Microsomal lipid peroxidation. Methods Enzymol.

[r8] Chehab M, Madala A, Trussell JC (2015). On-label and off-label drugs used in the treatment of male
infertility. Fertil Steril.

[r9] Chen A, Bookstein JJ, Meldrum DR (1991). Diagnosis of a testosterone-secreting adrenal adenoma by
selective venous catheterization. Fertil Steril.

[r10] Cherry N, Moore H, McNamee R, Pacey A, Burgess G, Clyma JA, Dippnall M, Baillie H, Povey A, participating centres of Chaps-UK (2008). Occupation and male infertility: glycol ethers and other
exposures. Occup Environ Med.

[r11] Cohen G, Dembiec D, Marcus J (1970). Measurement of catalase activity in tissue
extracts. Anal Biochem.

[r12] Elgawish RAR, Abdelrazek HMA (2014). Effects of lead acetate on testicular function and
caspase-3-expression with respect to the protective effect of cinnamon in
albino rats. Toxicol Rep.

[r13] Gabby PN (2006). Lead: in Mineral Commodity Summaries.

[r14] Garu U, Sharma R, Barber I (2011). Effect of lead toxicity on developing testis of
mice. Int J Pharm Sci Res.

[r15] Gorbel F, Boujelbene M, Makni-Ayadi F, Guermazi F, Croute F, Soleilhavoup JP, El Feki A (2002). Cytotoxic effects of lead on the endocrine and exocrine sexual
function of pubescent male and female rats. Demonstration of apoptotic
activity. C R Biol.

[r16] Gore AC (2001). Environmental toxicant effects on neuroendocrine
function. Endocrine.

[r17] Guay AT, Bansal S, Heatley GJ (1995). Effect of raising endogenous testosterone levels in impotent men
with secondary hypogonadism: double blind placebo-controlled trial with
clomiphene citrate. J Clin Endocrinol Metab.

[r18] Heinonen PK. (1991). Androgen production by epithelial ovarian tumors in
post-menopausal women. Maturitas.

[r19] Hernández-Ochoa L, García-Vargas G, López-Carrillo L, Rubio-Andrade M, Móran-Martínez J, Cebrián ME, Quintanilla-Vega B (2005). Low lead environmental exposure alters semen quality and sperm
chromatin condensation in northern Mexico. Reprod Toxicol.

[r20] Hsu PC, Liu MY, Hsu CC, Chen LY, Guo YL (1997). Lead exposure causes generation of reactive oxygen species and
functional impairment in rat sperm. Toxicology.

[r21] Jackie T, Haleagrahara N, Chakravarthi S (2011). Antioxidant effects of Etlingera elatior flower extract against
lead acetate-induced perturbations in free radical scavenging enzymes and
lipid peroxidation in rats. BMC Res Notes.

[r22] Kasperczyk S, Birkner E, Kasperzyk A, Zalejska-Fiolka J (2004). The activity of superoxide dismutase and catalase in people
protractedly exposed to lead compounds. Ann Agric Environ Med.

[r23] Katz DJ, Nabulsi O, Tal R, Mulhall JP (2012). Outcomes of clomiphene citrate treatment in young hypogonadal
men. BJU Int.

[r24] López-Carrillo L, Torres-Sánchez L, Garrido F, Papaqui-Hernández J, Palazuelos-Rendón E, López-Cervantes M (1996). Prevalence and determinants of lead intoxication in Mexican
children of low socioeconomic status. Environ Health Perspect.

[r25] Mattison DR (1983). The mechanisms of action of reproductive toxins. Am J Ind Med.

[r26] Misra HP, Fridovich I (1972). The role of superoxide anion in the autoxidation of epinephrine
and a simple assay for superoxide dismutase. J Biol Chem.

[r27] Naha N, Bhar RB, Mukherjee A, Chowdhury AR (2005). Structural alteration of spermatozoa in the persons employed in
lead acid battery factory. Indian J Physiol Pharmacol.

[r28] Naz RK (1999). Endocrine disruptors. Effects on Male and Female Reproductive
Systems.

[r29] Ng TP, Goh HH, Ng YL, Ong HY, Ong CN, Chia KS, Chia SE, Jeyaratnam J (1991). Male endocrine functions in workers with moderate exposure to
lead. Br J Ind Med.

[r30] NIH - National Institutes of Health (1988). Institutional Administrator's Manual for Laboratory Animal Care and
Use.

[r31] NRC - National Research Council-Institute for Laboratory Animal
Research (1996). Guide for the care and use of laboratory animals.

[r32] Patankar SS, Kaore SB, Sawaneh MV, Mishra NV, Deshkar AM (2007). Effect of clomiphene citrate on sperm density in male partners of
infertile couples. Indian J Physiol Pharmacol.

[r33] Petrusz P, Weaver CM, Grant LD, Mushak P, Krigman MR (1979). Lead poisoning and reproduction: effects on pituitary and serum
gonadotropins in neonatal rats. Environ Res.

[r34] Pinon-Lataillade G, Thoreux-Manlay A, Coffigny H, Masse R, Soufir JC (1995). Reproductive toxicity of chronic lead exposure in male and female
mice. Hum Exp Toxicol.

[r35] Ramos-Vara JA (2011). Principles and Methods of Immunohistochemistry. Methods Mol Biol.

[r36] Ribeiro RS, Abucham J (2009). Recovery of persistent hypogonadism by clomiphene in males with
prolactinomas under dopamine agonist treatment. Eur J Endocrinol.

[r37] Ring JD, Lwin AA, Köhler TS (2016). Current medical management of endocrine-related male
infertility. Asian J Androl.

[r38] Rönnberg L, Reinilä M Ylikorkala O (1985). Effects of experimental hyperprolactinemia and clomiphene on
pituitary responsiveness to LHRH and TRH in men. Andrologia.

[r39] Rose MP (1998). Follicle stimulating hormone international standards and
references preparations for the calibration of immunoassays and
bioassays. Clin Chim Acta.

[r40] Sanocka D, Kurpisz M (2004). Reactive oxygen species and sperm cells. Reprod Biol Endocrinol.

[r41] Shu J, Qiu G, Ilyas M (2014). Immunohistochemistry (IHC) Image Analysis Tool
box. ImageJ plugin.

[r42] Talalay P (1962). Hydroxysteroid dehydrogenases. Methods Enzymol.

[r43] Tannenbaum LV, Bazar M, Hawkins M, Cornaby BW, Ferguson EA, Chantelle Carroll L, Ryan PF (2003). Rodent sperm analysis in field-based ecological risk assessment:
pilot study at Ravenna army ammunition plant, Ravenna, Ohio. Environ Pollut.

[r44] Taskinen H, Lindbohm ML, Sallmén M, Gupta RC (2011). Occupational exposure to chemicals and reproductive
health. Reproductive and Developmental Toxicology.

[r45] Tenover JS, Bremner WJ (1991). The effects of normal aging on the response of the
pituitary-gonadal axis to chronic clomiphene administration in
men. J Androl.

[r46] Ulloa-Aguirre A, Timossi C (1998). Structure-function relationship of follicle stimulating hormone
and its receptor. Hum Reprod Update.

[r47] WHO - World Health Organization (2010). WHO laboratory manual for the Examination and processing of human
semen.

[r48] Wilhelm D, Palmer S, Koopmans P (2007). Sex detonation and gonadal development mammals. Physiol Rev.

